# Remodelling surgery with 3D printed patient specific surgical guides in patients with chronic diffuse sclerosing osteomyelitis/tendoperiostitis of the mandible, a case series

**DOI:** 10.4317/medoral.26410

**Published:** 2024-02-18

**Authors:** Marieke M van de Meent, Roy P J van den Ende, Sarina E C Pichardo, J P Richard van Merkesteyn

**Affiliations:** 1 MD, DDS. Resident maxillofacial surgery and PhD student at the Department of Oral and Maxillofacial Surgery, Leiden University Medical Centre, Leiden, the Netherlands; 2 MSc, PhD. Technical physician at 3D-Lab+ LUMC and at the Department of Oral and Maxillofacial Surgery, Leiden University Medical Centre, Leiden, the Netherlands; 3 MD, DDS, PhD. Maxillofacial surgeon at the Department of Oral and Maxillofacial Surgery, Leiden University Medical Centre, Leiden, the Netherlands; 4 DDS, MD, PhD. Professor and maxillofacial surgeon at the Department of Oral and Maxillofacial Surgery, Leiden University Medical Centre, Leiden, the Netherlands

## Abstract

**Background:**

Patients with chronic diffuse sclerosing osteomyelitis/tendoperiostitis (DSO/TP) of the mandible may complain about facial asymmetry as a result of mandibular deformity, one of the characteristics of DSO/TP of the mandible. If the disease is fully extinguished, remodelling surgery could be performed to treat complaints of facial asymmetry. This study reports the results of remodelling surgery with three-dimensional (3D) designed- and -printed patient-specific surgical guides.

**Material and Methods:**

3D printed guides were designed and manufactured by using mirroring of the contralateral non-affected mandible. Subsequently, the surgical procedure was performed under general anaesthesia using these surgical guides.

**Results:**

Four patients (all female) aged 15 (±2.8) years were included. They all complained about facial asymmetry and were planned for surgery with patient-specific surgical guides. Three of those surgeries were performed, of which two patients were satisfied with the result and the other patient is planned for re-surgery because of persistent aesthetical complaints. The last patient cancelled her surgery, because she eventually accepted her asymmetry with the help of a psychologist.

**Conclusions:**

The use of patient-specific surgical guides in remodelling surgery of the mandible could enable a more predicTable and symmetrical outcome, which could minimise the chance for re-surgery and could increase patient satisfaction. Furthermore, it could minimise the chance of iatrogenic damage to the inferior alveolar nerve.

** Key words:**Diffuse sclerosing osteomyelitis, chronic tendoperiostitis, pain, surgery, remodelling, 3D.

## Introduction

Chronic diffuse sclerosing osteomyelitis (DSO) or chronic tendoperiostitis (TP) of the mandible is a rare disease with high morbidity and abundant use of painkillers. The disease is characterised by recurrent pain and swelling of the cheek often accompanied by trismus (1-5). The aetiology and pathogenesis of the disease is still debated. Since the lesions are particularly located around the attachment sites of the masticatory muscles, it is hypothesised that it could arise from overactive use of the masticatory muscles, also known as chronic tendoperiostitis (6-8).

Progressive growth of the mandible is observed in some of these patients. This specific feature for DSO/TP could be caused by remodelling of the bone, as a reactive response to external forces of the masticatory muscles (9). Even if disease activity is fully extinguished, mandibular deformity may persist. In some patients, aesthetic complaints may arise. After ruling out the activity of the disease, a remodelling mandibular surgery could be considered.

The aim of this study is to report the results of remodelling surgery of the mandible with three-dimensional (3D) printed patient-specific surgical guides in patients with aesthetic complaints of mandibular deformity caused by DSO/TP of the mandible.

## Material and Methods

Consecutive patients with DSO/TP of the mandible who presented themselves for follow-up consult at the Department of Oral and Maxillofacial Surgery, Leiden University Medical Center (LUMC), Leiden, the Netherlands and complained about facial asymmetry due to deformity of the mandible, were included in this study. Each included patient gave consent for participation.

- Preoperative planning, design and manufacturing of individualised 3D guiding templates

If a patient with DSO/TP complained about facial asymmetry during follow-up consult, the disease activity was assessed. A remodelling mandibular surgery could be considered if the disease was fully extinguished, which means that the patient was symptom-free for at least six months (no swelling, trismus and/or pain) and no disease-activity on radiographs was seen.

A full skull cone beam computed tomography (CBCT) scan (Planmeca Promax®3D Max, reconstructed slice thickness 0.4 mm, pixel spacing 0.4 mm x 0.4 mm, 96 kVp, tube current 5 mA) was performed for preoperative planning of the surgery. The scan volumes were exported in DICOM format and imported in Mimics (Materialise, Leuven, Belgium). Using threshold segmentation, a mask of the mandible was created and converted to a standard tessellation language (STL) file. Subsequently, the STL file of the mandible was imported in 3-Matic (Materialise, Leuven, Belgium). The unaffected side of the mandible was mirrored to determine the extent of the asymmetry on the affected side. Then the resection osteotomies were determined, aiming for a close match with the healthy unaffected side, with regard to the alveolar nerve.

Subsequently, a patient-specific surgical guide was designed that indicated the planned osteotomy and these were bone or dentition supported. The surgical guides were produced from polyamide using selective laser sintering and sterilized before surgery. It was important to make the surgical guide as small as possible, in order to enable predicTable placement of the guide.

Patients were referred to the endocrinologist for intravenous bisphosphonate therapy prior to and/or after surgery to ensure a low bone metabolism peri- and post-operatively.

- Surgical procedure

The surgical procedure was performed under general anaesthesia. After intubation, the surgical area was infiltrated with epinephrine (Ultracaine D-S, Aventis Pharma, Hoevelaken, the Netherlands) in order to reduce bleeding and maximise visibility of the surgical field. A relatively large incision was made in the mucosa, at least 3mm below the attached gingiva, and extension of the incision was often performed as far as necessary. A periosteal elevator was used to subperiosteally prepare the affected mandibular region. The mental foramen was visualised if this was included (or close to) the affected region.

PredicTable placement of the patient-specific surgical guide was of vital importance to obtain predicTable results. Limited flexibility of the soft tissues and/or presence of important anatomical structures could complicate predicTable placement of the surgical guide. These factors were therefore accounted for in both the design of the surgical guide (as small as possible), and the incision (with sufficient subperiosteal elevation to place the guide). The surgical guide was placed and fixed to the mandible using either an occlusion-based splint that was attached to the guide, or using several placement screws. Subsequently, the patient-specific 3D-printed surgical guide predicted the predetermined (3D-planned) resection osteotomies. This ensured that as much (excessive) bone could be removed and therefore as much facial symmetry as possible could be achieved, without damaging the mandibular nerve and/or other vital structures.

The osteotomies that allowed relatively easy access were performed using a saw, whereas the (parts of) the osteotomies that were difficult to access were performed using a piezotome with different cutting tips. If necessary, chisels were used to complete the osteotomies. Subsequently, after enough bone was removed, the sharp bone edges were smoothened using a burr.

All wounds were primarily closed and an extra-oral pressure bandage was applied.

- Postoperative management

Patients were admitted to the hospital for a short stay of 2-4 days for postoperative pain management. Antibiotics were routinely administered peri-operatively and for 24-48 hours after surgery to prevent infection, also because of bisphosphonate use.

After surgery, a CBCT-scan was performed to assess the result of the surgery. Patients were seen for routine check-ups postoperatively and check-ups for management of DSO/TP.

## Results

This case series included four patients, of whom all were female. Demographic and clinical characteristics of the patients are listed in Table 1. The mean (±SD) age at the time of diagnosis of the disease was 15 (±2.8) years. In two patients DSO/TP was located in the right mandible, and in the other two patients it was located in the left mandible.

**Figure 1:**
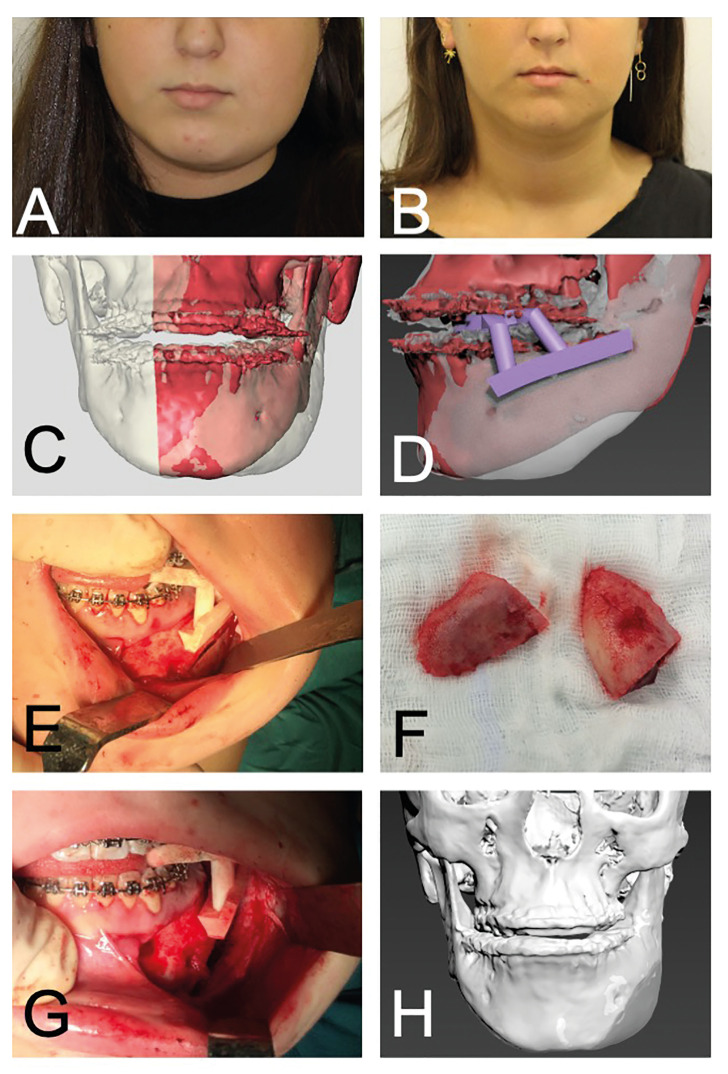
A 13-year-old patient with DSO/TP of the left mandible. (A + B) Pre- and post-operative pictures, on which a decrease in swelling of the left mandible can be observed. (C + D) Three-dimensional reconstruction (3D) of CBCT-scan on which the osseous swelling of the left mandible can be seen. In red a mirrored model of the mandible to determine the extent of asymmetry and to determine the resection osteotomy with matching patient-specific surgical guides. (E) Peri-operative image after incision with visualization of the mental foramen and placement of the occlusion-based patient-specific surgical guide. The osteotomy-lines prepared with a saw can be seen. (F) The removed bone parts. (G) Picture taken intra-orally after removing the bone parts with the patient-specific surgical guide still inside. (H) 3D reconstruction of CBCT-scan pre- (transparent) and postoperative, showing a more symmetrical appearance.

The first patient was a 13-year-old girl at the time of first presentation. Her complaints started after the start of orthodontic treatment and consisted of recurring pain and perimandibular swelling of the left mandible. Panoramic radiograph and CT-scan showed mixed sclerosis and osteolysis of the mandibular bone on the left side, and bone scintigraphy showed intense uptake of radiopharmacon of the left mandible. A bone biopsy was performed in the referring hospital, which showed a reactive periosteal bone-forming process, with multiple lucencies, consistent with chronic osteomyelitis. She had already been treated with several courses of antibiotics without result. After referral she started with conservative therapy consisting of occlusal splint therapy, counselling about the disease and physical therapy (with habit reversal training, myofeedback and relaxation therapy). Her pain complaints improved, but mandibular asymmetry persisted. Therefore, she underwent a conventional remodelling resection of the left mandible twice at the age of 16 years with intravenous bisphosphonates three months prior to surgery. Six months after the last surgery, she still complained about facial asymmetry. Given the location of the osseous asymmetry and the location of the alveolar nerve, a remodelling surgery with 3D printed patient specific guides seemed possible (Fig. 1).

**Table 1:**
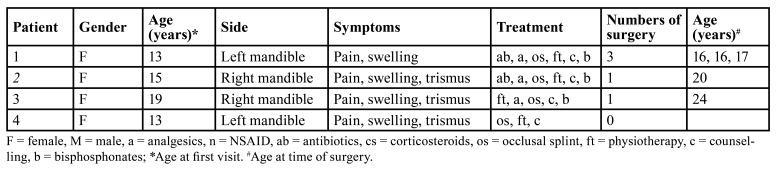
Demographic and clinical characteristics of patients.

The surgery was performed when she was 17 years old. After surgery she had no neurosensory disturbances of the lower lip and she was satisfied with the result.

**Figure 2:**
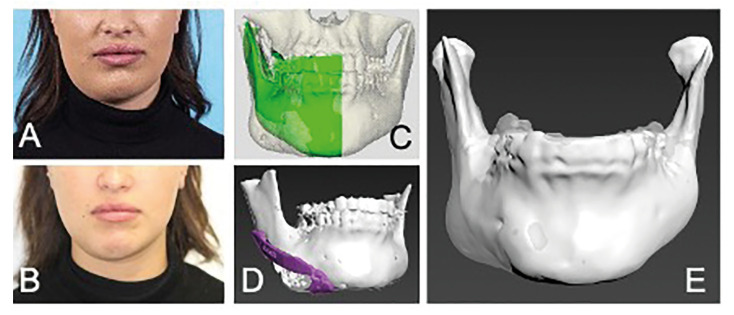
A 15-year-old patient with DSO/TP of the right mandible. (A + B) Pre- and post-operative pictures, on which a decrease in swelling of the right mandible can be observed. (C + D) 3D reconstruction of CBCT-scan on which the osseous swelling of the right mandible can be seen. In green a mirrored model of the mandible to determine the extent of asymmetry and to determine the resection osteotomy with matching patient-specific surgical guides. (E) 3D reconstruction of CBCT-scan pre- (transparent) and postoperative, showing a more symmetrical appearance d.

The second patient was a 15-year-old girl with complaints of recurring pain, swelling of the right mandible and trismus. She already had complaints for five years when she was referred and was already treated with several courses of antibiotics and analgesics. Panoramic radiograph, CBCT-scan and bone scintigraphy showed diffuse osteosclerosis and -lysis of the right mandible, and increased uptake of the right, but also the left mandible suiTable for the diagnosis chronic diffuse sclerosing osteomyelitis. After referral she started with the aforementioned conservative treatment. After four years she indicated the desire for a remodelling resection. She underwent remodelling surgery (when she was 20 years old) with 3D printed patient specific guides (Fig. 2) with intravenous bisphosphonates prior to and after surgery. After surgery she had transient neurosensory disturbances of the lower right lip. At first, she was satisfied with the result, however, after two years she complained about renewed facial asymmetry again, for which she is planned for a second remodelling surgery with patient specific guides.

**Figure 3:**
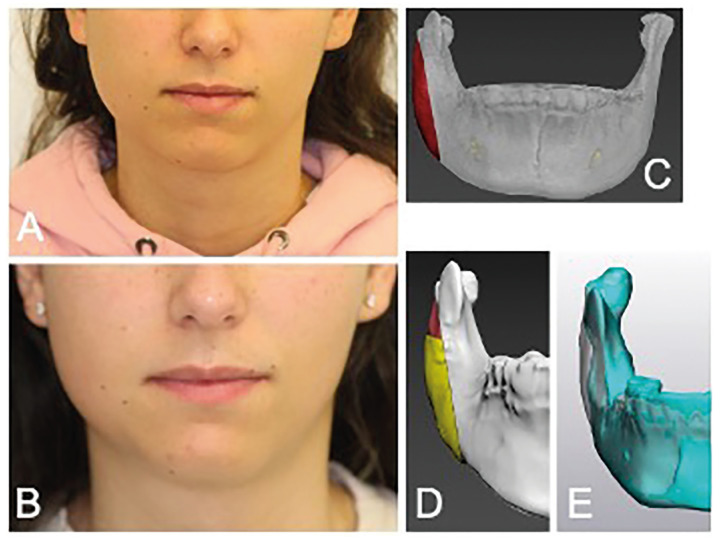
A 19-year-old patient with DSO/TP of the right mandible. (A + B) Pre- and post-operative pictures with a decrease in asymmetry on the right side. (C + D) Three-dimensional reconstruction (3D) of CBCT-scan on which the osseous swelling can be seen, with a planning for remodelling surgery. In red a mirrored model to determine the asymmetry and to determine the resection osteotomy, and in yellow the matching patient-specific surgical guide. (E) 3D reconstruction of the pre- (transparent) and postoperative (blue) situation, showing a more symmetrical appearance.

The third patient was a 19-year-old female with complaints of recurring pain, trismus, and swelling of the right mandible for six years. She was treated with orofacial physical therapy, removal of the third molar of the right mandible without result, and several types of analgesics. Panoramic radiograph, CBCT-scan and bone scintigraphy showed subperiosteal bone formation, condylar process deformation, osteosclerosis and increased uptake of the right mandible. After referral she started with the aforementioned conservative therapy, however, because of little therapy adherence she still had complaints and was referred to the endocrinologist for bisphosphonate therapy. After intravenous bisphosphonate therapy, she reported improvement of her pain complaints and the panoramic radiograph improved. Only complaints of facial asymmetry remained. Her surgery was postponed, because of renewed complaints. After treatment with intravenous bisphosphonates and a complaint-free period of six months, she underwent remodelling surgery with 3D printed patient specific guides, when she was 24 years old (Fig. 3).

After surgery she had no neurosensory disturbances of the lower lip and she was satisfied with the result.

The last patient was a 13-year-old girl with recurring pain, trismus and facial asymmetry on the left side for two years. Panoramic radiograph and CBCT-scan showed diffuse sclerosis of the left mandible with alternating osteolysis of the body of the mandible. She started with conservative therapy, with good results. After five years she indicated the desire for a remodelling resection. However, she cancelled her surgery, because she eventually has accepted her appearance with the help of a psychologist.

## Discussion

This study shows that remodelling surgery in patients with DSO/TP of the mandible and complaints of facial asymmetry could be performed making use of 3D printed templates. The surgical guide takes into account the alveolar nerve, and mirrors the contralateral healthy side to pursue facial symmetry.

This case series described techniques for remodelling surgery in patients with DSO/TP of the mandible, that experienced residual mandibular deformity (i.e. facial asymmetry) after their DSO complaints had been successfully treated using non-surgical treatment options (conservative treatment and/or bisphosphonate therapy) (7,8,10). The patients in this case series had therefore been free of DSO complaints for more than six months.

In our view (and in our treatment protocol), there is no place for surgery in the first phase of treatment of DSO/TP of the mandible with complaints. Previous studies have shown that (invasive) surgical treatment to cure DSO complaints has a low rate of success and high risk of morbidity, such as the risk of damaging the inferior alveolar nerve, deformity, problems with mandibular reconstruction and -growth (in children) (11). In our view, surgery is therefore contra-indicated in patients that still experience complaints and/or show progressive deformity. However, if patients are treated successfully with treatments, such as analgesics, conservative/non-surgical therapy, intravenous bisphosphonates, or a combination of these non-surgical treatment options, remodelling surgery could be an option to correct residual mandibular deformity. This remodelling surgery is, thus, only performed for aesthetic purposes and only if the disease is fully extinguished.

As described in these cases, at first, performing mandibular remodelling surgery was started conventionally, without patient specific guides. However, this was a difficult procedure, because it is difficult to assess symmetry during surgery, especially at the lower border of the mandible and peri-operatively it is difficult to determine how much of the mandible can be resected without causing damage to the inferior alveolar nerve. Therefore, with the ability to create 3D patient specific guides, defining the resection margins using the contralateral side of the mandible and taking into account the inferior alveolar nerve, we started to perform this remodelling surgery with 3D preparation and 3D printed templates. This renewed approach could prevent and minimise iatrogenic damage to the inferior alveolar nerve, and could obtain a better predicTable facial symmetry, and therefore more predicTable surgical results (12). The soft tissues and increased convexity of the lower border make it hard to reach the lower border of the mandible, as was experienced when performing the surgery in case 1 and 2. However, if the template did not fit, because of the soft tissues, it could indicate approximately how much of the mandible had to be resected. Given the aesthetic indication for this surgery, an extra-oral approach was not considered, given the possible complications (such as nerve damage to the marginal mandibular branch of the facial nerve) and a (possible) visible scar in the neck that this approach causes.

The surgery should be postponed if patients had symptoms (swelling, trismus, and/or pain) the previous year and/or if disease activity was seen on radiographs (as occurred in the third patient). In case of disease activity, active bone turnover is expected, which can cause bone growth and again mandibular deformity. Even at a later time, the disease can flare up and thus bone turnover can be seen again. Therefore, all operated cases received intravenous bisphosphonates prior to and after surgery to ensure a low bone metabolism peri- and post-operatively. Bisphosphonates inhibit osteoclasts, which are involved in bone resorption, -turnover, and -renewal, and therefore it decreases those processes (10,13-17). Also, for the treatment of DSO/TP of the mandible bisphosphonates seem to be successful, with high chance of remission of symptoms (7,10,13-23).

In summary, we believe that remodelling surgery in patients with DSO/TP of the mandible and complaints about facial asymmetry should be performed by using a 3D designed- and -printed patient-specific surgical guide. Nevertheless, possible disadvantages should be considered. Designing and manufacturing these individualised surgical guides is associated with additional costs. However, we think that these costs can be compensated by the fact that the surgical time could be shorter, the chance for re-operation could be less, and the (aesthetical) results, and thus patient satisfaction, could be better (24). In future, it could be of interest to study the surgical time, re-operation and patient satisfaction using a 3D designed- and printed patient-specific surgical guide compared to the conventional surgery. However, it is difficult to reach bigger sample sizes of patients with DSO/TP of the mandible, since it is a rare disease.

## Conclusions

The use of a 3D designed- and -printed patient-specific surgical guide in remodelling surgery of the mandible in patients with DSO/TP of the mandible and complaints of facial asymmetry could enable a more predicTable surgery. Preparation of saw osteotomies with an optimal placement of surgical guides is facilitated by this technique, which could minimise the chances of iatrogenic damage to the inferior alveolar nerve and could result in more predicTable and symmetrical outcomes. This could minimise the chance for re-surgery and it could increase patient satisfaction.
